# Workplace Bullying as a Risk Factor for Musculoskeletal Disorders: The Mediating Role of Job-Related Psychological Strain

**DOI:** 10.1155/2015/712642

**Published:** 2015-10-18

**Authors:** Michela Vignoli, Dina Guglielmi, Cristian Balducci, Roberta Bonfiglioli

**Affiliations:** ^1^Department of Psychology, Alma Mater Studiorum-University of Bologna, Viale Berti Pichat 5, 40127 Bologna, Italy; ^2^Department of Educational Science, Alma Mater Studiorum-University of Bologna, Via Filippo Re 6, 40126 Bologna, Italy; ^3^Department of Political and Social Sciences, Alma Mater Studiorum-University of Bologna, Via dei Bersaglieri 6/c, 40125 Bologna, Italy; ^4^Occupational Medicine, Department of Medical and Surgical Sciences, Alma Mater Studiorum-University of Bologna, Via Palagi 9, 40138 Bologna, Italy

## Abstract

Workplace bullying is considered by the European Agency for Safety and Health at Work one of the emerging psychosocial risk factors that could negatively affect workers' health. Thus, the aim of this study was to analyze the process that leads from bullying to negative health (such as musculoskeletal disorders (MSDs)), testing the mediating role of job-related strain. Data were collected on 512 workers (62.9% female; mean age = 43.6 years) of a retail chain who filled in a self-report questionnaire after a one-hour training session on work-related stress. Data analyses were performed controlling for potentially confounding variables (i.e., gender, age, organizational role, type of contract, and perceived physical job demands). Preacher and Hayes analytical approach was used to test the indirect relationship between bullying and MSDs. Results showed that work-related strain mediates the relationship between bullying and MSDs considered (low back, upper back, and neck) except for MSDs of the shoulders. Our study confirms the role played by bullying and job-related strain in determining workers' MSDs.

## 1. Introduction

Increasing attention has been paid in the past 15 to 20 years to the phenomenon of workplace bullying; in some countries it is also called mobbing [1]. Workplace bullying refers to a series of negative behaviours carried out frequently and over a prolonged period of time, usually against an individual employee by his or her colleagues or superior [2]. Examples of such negative behaviours are as follows: excessive criticism of one's work; withholding of information, which affects performance; being assigned an unmanageable workload; spreading of rumours; and social isolation.

Bullying is an escalating process in the course of which the person confronted ends up in an inferior position and becomes the target of systematic negative social acts. Therefore, a conflict cannot be called bullying if the incident is an isolated event or if it involves two parties of approximately equal strength [2]. The consequences of exposure to bullying may be traumatic for the affected individual [3, 4]. Prevalence estimates of bullying are difficult due to a lack of an agreed upon definition of the phenomenon. A recent European survey [5] estimated a prevalence of 4% among European workers. However, in the same survey, 11% of workers reported they were the subject of verbal abuse at work, which may also be considered a form of bullying. According to others, the prevalence of bulling may be even higher: 15% of workers may be affected at any point in time [6]. Despite this lack of convergence on prevalence estimates, there is substantial agreement that workplace bullying is an emerging psychosocial risk with the potential to adversely affect the safety and health of working people [7].

Most studies in this area have investigated psychological health outcomes of exposure to bullying, documenting a significant relationship between bullying and psychosocial stress, leading to anxiety and depression, including the onset of major depressive episodes [8–12]. It is now quite clear that exposure to bullying can lead to a profound deterioration of the person's psychological health, mainly via stress experiences [13]. Few studies, however, have investigated the potential impact of bullying on outcomes other than psychological ones. Thus, it remains to be seen whether bullying has the same far-reaching health effects as those, for example, of well-established psychosocial factors, such as job strain or effort-reward imbalance, which have been found to deteriorate not only to psychological but also to physical health conditions [14]. Furthermore, researchers have noted that studying the relationship between psychosocial factors, such as bullying, which are usually assessed through self-reports, and psychological outcomes, may be particularly subjected to common method bias due to personal factors such as negative affectivity, which may act as a critical confounding variable [15]. This further strengthens the relevance of assessing the potential effect of bullying on different kinds of health-related outcomes.

To address the gap in the literature presented above, in the present study we investigate the relationship between exposure to bullying and very common work-related physical health problems, namely, musculoskeletal disorders (MSDs). MSDs are dysfunctions affecting muscles, bones, nerves, tendons, ligaments, joints, cartilages, and spinal discs; they are defined by sprains, strains, tears, soreness, pain, peripheral nerve disorders, and connective tissue injuries of the structures previously mentioned [16]. MSDs are the most often reported health problem by workers in the European Union: 24.7% of them report back pain and 22.8% report muscular pain in shoulders, neck, upper or lower limbs, or combinations of any or all of these. In the United States, MSDs are one of the main reasons for short- and long-term disability and early retirement [17, 18].

The most common antecedents of MSDs are biomechanical factors, such as repetitive motion, excessive force, awkward postures, and prolonged sitting and standing [16]. However, psychosocial factors are also believed to be important for both the initial development of MSDs and the long-term disability that may follow [18–22]. While the precise mechanisms (e.g., cognitive, neuroendocrine, and musculoskeletal) through which psychosocial factors may affect MSDs have not been fully elucidated, an accepted hypothesis [23] is that psychosocial factors may operate indirectly. They may, for example, influence muscle tension or other physiological processes and decreasing micropauses in muscle activity and, as a consequence, affect the perception of pain. Plausibly, such indirect effect is exerted through the experience of work-related stress.

Most research on the impact of psychosocial factors on MSDs has focused on factors, such as psychological job demands and job control [24]. A review of the available evidence suggests that such factors (i.e., high demands and low control) are indeed related to MSDs, specifically of the neck, shoulder, and back [25]. As far as exposure to bullying is concerned, we traced two studies exploring its relationship with MSDs. A study on 370 Lithuanian seafarers published as a conference abstract revealed that exposure to bullying was significantly associated with an overall measure of upper limb MSDs [26]. Another study conducted on 1024 employees of a Norwegian bus company revealed an association between exposure to bullying and a measure of musculoskeletal complaints including headache, backache, neck ache, and hand and foot pain [27]. However, the latter study did not control for potentially confounding factors, such as physical load factors. Furthermore, neither study followed recent recommendations emphasizing the importance of investigating specific forms of MSDs [25].

Thus, in the present study, we further investigate the relationship between exposure to bullying and MSDs by controlling for potentially confounding factors and focusing on specific musculoskeletal problems. Furthermore, we explore whether job-related strain may indeed act as a mediator in the relationship between exposure to bullying and MSDs, as Silverstein and Evanoff [23] hypothesized and, indeed, as Sprigg et al. [24] found for other psychosocial risk factors.

## 2. Methods

### 2.1. Study Design and Sample

A cross-sectional survey was conducted in a large retail company in Italy. A total of 553 workers voluntarily participated in the study, after researchers obtained a randomized sample from the organization's 812 workers (68.1% was the response rate). All participants worked in grocery stores belonging to the same organization; therefore all of them have the same procedures and company regulations. The sample was composed of both supervisors and employees. Participants worked in different departments of the supermarkets (e.g., gastronomy, fruit and vegetables, butchery, fish, bakery, cashiers, and nonfood); thus they all perform job activities with high physical demands.

Workers were assembled in different groups and, after one hour of training on work-related stress, they completed an anonymous, self-administered questionnaire. The contents of this brief training session were the main European and national regulations about work-related stress and the main definitions of work-related stress used in the literature. This training hour was included before filling the questionnaire in order to explain to the workers that the aim of the study was not to define how much they were stressed, but only to understand which psychosocial risk factors could contribute to enhancing strain and decreasing workers' health.

### 2.2. Measures

Workplace bullying is normally assessed either by using the respondents' feeling of being victimized by bullying (e.g., [9]), usually according to a given specific definition of the phenomenon, or according to the respondents' perception of being exposed to a range of specific bullying behaviours described without explicit reference to the term* bullying* (e.g., [28]). The first method is the so-called self-labelling approach, which, however, is very subjective and strongly influenced by personality and emotional and cognitive factors, including possible misperception. The second method is the behavioural experience method, which is generally believed to be more objective because it is relatively less exposed to the effect of personal factors. Thus, in the present study, we used the latter approach and assessed bullying with the Italian version of the Short Negative Acts Questionnaire (S-NAQ) [29], which has been validated in Italy with an ad hoc study [30]. The scale consists of 9 items investigating how often the respondent has experienced a variety of negative behaviours at work during the last six months. One example item is “Someone withholding information, which affects your performance” and workers could answer on a 5-point Likert scale ranging from 1 (*never*) to 5 (*daily*). Items were then averaged. The S-NAQ has shown psychometric properties using Italian data, which are entirely comparable to those of the original and longer (i.e., 22-item) version, for example, in terms of associations with variables of mental health and well-being [30].

Job-related strain was measured through the dimension of emotional exhaustion of the Maslach Burnout Inventory General Survey (MBI-GS: [31]; Italian version [32]). The 5-item scale was scored on a 7-point frequency Likert scale (0 =* never* to 6 =* every day*). One example item is as follows: “I feel emotionally drained from my work.” Items were then averaged.

Musculoskeletal disorders were measured through 4 items related to four different parts of the body: low back, upper back, neck, and shoulders. The question was, “During the past 12 months have you had pain, aching, stiffness, burning, numbness, or tingling (“pins and needles”) in any areas of the following that occurred more than three times or at least more than a week?” The possible answers were either* yes* or* no*.

In addition to those variables, possible confounding variables were included: gender, age, organizational role, type of contract. Furthermore, as participants were working in a large retail company, we introduced physical job demand measured with the Italian version [33] of Karasek's [34] Job Content Questionnaire as a control variable. The scale consists of 5 items with response options ranging from 1 (*strongly disagree*) to 4 (*strongly agree*). One example item is “I am often required to move or lift very heavy loads on my job.” Items were then averaged.

### 2.3. Statistical Analysis

Logistic regression models were fitted to the data by using the software SPSS version 20.0. The risk factor was bullying, while the outcome variables were four specific MSDs of the low back, upper back, neck, and shoulders. To test for the mediating role played by job-related strain (i.e., emotional exhaustion) in the relationship between exposure to bullying and MSDs, we adopted the Preacher and Hayes [35] analytical approach. This approach tests the indirect relationship between an exposure factor and an outcome through a mediator by using a bootstrap (i.e., resampling) procedure that addresses some weaknesses associated with the Sobel test [35]. To compute the direct and indirect effects, all path coefficients in the model were estimated concurrently. Furthermore, the bootstrapping procedure was used to compute formal statistical tests of the specific indirect effects. This method can produce an estimate of the indirect effect, including a 95% confidence interval. When 95% confidence interval does not include zero, the indirect effect is significantly different between the level of zero and 0.05. Four different mediation analyses were performed, one for each specific MSD, that is, for the low back, upper back, neck, and shoulders.

## 3. Results

### 3.1. Demographic and Working Characteristics of Subjects

Due to missing data, 41 cases were deleted; thus, the final sample consisted of 512 Italian workers. Most of them (322 workers, 62.9%) were female and the mean age was 43.64 years (SD = 7.8). The mean occupational tenure was 16.15 years (SD = 8.46). Concerning the type of contract, 52.3% had a part-time contract, while all other workers had a full-time contract. Concerning the organizational role, 94 workers (18.4%) were supervisors, while 418 were employees (81.6%).

### 3.2. Descriptive Statistics, Correlations, and Job-Related Strain Mediation Effect between Bullying and MSDs

Means, standard deviations, percentages, internal consistencies, and correlations were computed for all the study variables (Table 1). Internal consistencies (Cronbach's *α*) of the used scales were good, as all the values exceeded the threshold of 0.70 [36]. Exposure to bullying behaviours was relatively low, meaning that, on average, employees only occasionally experienced those negative acts that are the essence of bullying (Table 1). The obtained value of 1.67 at the bullying measure is similar to that commonly found in organizational research in this area in which the same operationalization of bullying is used [37, 38]. A closer inspection of the distribution of the bullying variable revealed that 3.51% of employees (not reported in Table 1) reported a score indicating an exposure on a weekly or daily basis to the bullying behaviours investigated.

On the contrary, job-related strain and physical demand were relatively more prevalent, with their average levels (i.e., 17.30 and 2.71, resp.) being above the central point of the adopted response scale. For example, a score of 2.71 at the physical demand scale meant that all the five investigated aspects describing a high physical demand tended to be reported by most of participants. As far as musculoskeletal problems are concerned, in general they were highly prevalent among participants, with the highest prevalence being for the low back problems.

Furthermore, results, presented in Table 1, showed that, among the confounding variables (age, gender, organisational role, type of contract, and physical demands), all of them were related to at least one of the outcome variables considered (MSDs of low back, upper back, neck, and shoulders). Thus, these confounding variables have been included in the mediation analysis.

In order to test our hypothesis, which postulates that strain mediates between bullying and MSDs, four mediation analyses have been performed. As mentioned before, the Preacher and Hayes [35] analytical approach allowed us to test the direct and indirect effects of the variables considered. Thus, we provided estimates of all the path coefficients (Table 2), as well as indirect effects (Table 3) along with the 95% bias-corrected, bootstrapped confidence intervals for the four different musculoskeletal disorders (low back, upper back, neck, and shoulders). Specifically, in Table 2 both results concerning the direct effects of the antecedent and confounding variables on the mediator (job-related strain) and results concerning the direct effects of the antecedents, confounding variables, and the mediator on the outcomes (MSDs of low back, upper back, neck, and shoulders) are presented.

Thus, concerning the direct effects, bullying has a positive effect on strain and on all the MSDs considered, except for MSD of the shoulders. This means that the more workers are exposed to bullying, the more they report MSDs of the low back, upper back, and neck. Also, work-related strain is directly related to all MSDs, except for shoulders. Looking at the possible confounding variables, perceived physical demand has an effect both on strain and on all MSDs, while age affects strain and only MSD of the shoulders. Regarding gender, females report more MSDs but not higher strain. Organizational role and type of contract seem to not have an effect on either strain or MSDs.

Results concerning the indirect effects between the independent variable (bullying) and the outcomes variables (MSDs of low back, upper back, neck, and shoulders) are presented in Table 3. Results show that job-related strain mediates the relationship between bullying and all MSDs, except for MSDs of the shoulders. Those results mean that, except for the MSD of shoulders, strain helps in understanding the process between bullying and musculoskeletal disorders, as results presented in Table 3 show that bullying affects strain which in turn affects MSDs (low back, upper back, and neck).

## 4. Discussion

Even though psychosocial risk factors have been found to be implicated in the development of MSDs (see, for a review, [20]), most studies in this area have been inspired by Karasek et al.'s [39] psychosocial model and have investigated the role of psychological job demand (i.e., workload) and decision latitude (i.e., job control) on MSDs [24]. Having to do with the tasks performed by the worker, job demands and decision latitude are typical job content factors (see European Agency for Safety and Health at Work [40]). Psychosocial contextual factors, such as those describing the quality of relationships at work, have rarely been examined in detail. As far as workplace bullying is specifically concerned, only a few studies have explored the relationship between exposure to such contextual factors and MSDs [26, 27]. However, such studies have not adopted a fine-grained approach on MSDs or included an overall index of MSDs, which is less informative and generally not recommended [25]. Furthermore, there is a substantial lack of knowledge about the possible mechanisms for explaining the link between psychosocial factors and MSDs. The experience of psychological strain has been hypothesised as one such mechanism [23], but its involvement has rarely been directly explored.

Our results confirm that exposure to bullying behaviour is linked to MSDs (in the low back, upper back, and neck regions). Only the shoulders do not seem affected by this mediation. The results suggest that, along with the direct effect between bullying and MSDs (low back, upper back, and neck), there is a process which comprises job-related strain between workplace bullying and MSDs. Therefore this relationship ought to be explained by both the direct effect of bullying as a psychosocial factor and the indirect effect of psychological strain manifesting as MSDs. Furthermore, despite physical demands remaining the main predictor of MSDs, when strain is considered, the effect of bullying on MSDs is quite similar (especially on the basis of the upper back and neck).

Seeing that exposure to bullying can lead to a profound deterioration of the victim's psychological health mainly via the experience of stress [13], the same mechanism seems to also influence physical health, specifically MSDs. Formerly Vie et al. [27] found both positive and negative emotions mediate the relationship between exposure to bullying and musculoskeletal complaints, even if it seems that negative emotion, namely, stress, is the main mediator. In line with this study, to our knowledge, this is the first direct evidence of job-related strain as a mediator between bullying and MSDs. Therefore, the strain process, which notoriously may affect the body, for example, by producing tension in the musculature, is one of the elements to consider as we comprehend the detrimental effects of bullying on the victims' health. Note that we only found evidence for a partial mediation by psychological strain, since in the three cases had psychological strain acted as a mediator (i.e., of pain in the low back, upper back, and neck), bullying would have remained a significant risk factor for the investigated MSD in the final model.

One explanation for the direct effect between bullying and MSDs could be that we operationalized psychological strain in terms of emotional exhaustion, which mainly taps low-arousal symptoms, such as feelings of fatigue and depression, thus capturing only certain kinds of manifestation of psychological strain. High-arousal symptoms such as anxiety and irritability, which are not well represented in the emotional exhaustion construct, may be even more critical in mediating the effect of bullying on MSDs. This is because bullying has been shown to generate strong feelings of anxiety and, eventually, disorders in those who are exposed [3]; at the same time, anxiety has been found to be one of the stronger affective mediators of the relationship between psychosocial aspects of work and MSDs [41]. In brief, there is room to believe that the psychological strain generated by exposure to bullying may have an even more important role in the occurrence of MSDs than that found in the present study. This suggests the need for more research in this area.

One of the main strengths of this study is the focus on workplace bullying as a psychosocial risk factor for MSDs. Even though NIOSH [16] considers these health complaints an important occupational disease, relative to other psychosocial risk factors, they are still understudied. Another strong point is represented by the fact that work characteristics, workplace bullying, stress, and MSDs are studied together. Usually, the relationships between work characteristics, bullying, and stress find evidence in stress or psychological literature, whereas the relationships between work characteristics and MSDs are predominantly found within the medical, ergonomic, and epidemiological fields [24].

These strong points, however, do have some limitations that should be mentioned. First, the sample was not representative of a working population or of workers in the retail sector, which might decrease the opportunity to generalize the obtained results. A second limitation of the present study is that it is cross-sectional, so we cannot strengthen the basis for causal inference regarding MSDs. Therefore, adopting a rigorous longitudinal research design would reduce the likelihood of the findings having arisen due to chance and would allow us to investigate the effective impact that bullying has on workers who develop MSDs. Moreover, the adopted measures were paper-and-pencil reports, which can lead to biased responses from the subjects. Although adopting MSDs self-report represents a limitation, evidence suggests that questionnaires are more sensitive indicators of MSD problems than preexisting data sources [42]. However, in this study, objective measures would be suitable only for assessing the MSDs, for instance, by medical evaluation. On the other hand, attempting to collect objective measures of the presence of bullying in the workplace would not be feasible, due to problems linked to the measures of negative activities, such as bullying, which are subjective and difficult to identify [43]. Furthermore, it is not possible to state whether the training session could have partly impacted the workers' response rate but that session was considered necessary also from the company management as workers had to answer to questions concerning their health and potential issues concerning bullying at work. A final limitation is that the adopted measure of workplace bullying insisted exclusively on repetitive and prolonged exposure to negative workplace behaviours, thus ignoring other important defining elements of the bullying definition such as the perceived imbalance of power between target and perpetrator(s). Although measures insisting on exposure to negative acts are often used in the literature and they are also recommended when the aim of the study is to look at the relationship between bullying and other variables [44], such measures are far from being a perfect operationalization of bullying. Despite these limitations, the current findings have implications for future research directions and for practical implications. Indeed, for future studies on psychosocial risk factors and MSDs it may be interesting to investigate not only job demands, specifically workload and lack of autonomy, which are often studied as psychosocial risk factors associated with MSDs [45, 46], but also perceptions of work life quality and relationships within the workplace. In this study, initial outcomes of such relationships have been reported, although further study is needed not only pertaining to workplace bullying, but relative to the wider category of psychosocial contextual factors (i.e., role clarity, work-family conflict). Until now, these have not been studied in relation to MSDs, yet they are known to affect health. Moreover, future research should also investigate the reciprocal relationship between bullying, job-related strain, and MSDs.

Regarding practical implications, our results underline that, in addition to more traditional prevention strategies used to diminish biomechanical risk factors, establishing prevention strategies to reduce the presence of psychosocial risk factors, in particular, workplace bullying, in the organization of work should also be considered. Also, the mediating role of job-related strain suggests that the good practices mentioned above relative to ergonomic characteristics in the workplace cannot be decisive in solving the issue. When addressing MSDs, both biomechanical and psychological sources should be included. Our results, therefore, show that bullying can be the initiator of the process which could lead to an increase of MSDs, indicating the need to promote primary prevention intervention in the workplace to reduce bullying and, as a concequence, decrease perceived job-related strain and MSDs. Diverse studies have confirmed the role of organizational factors affecting bullying, such as perceived cognitive, emotional and behavioral social support from colleagues [47], perceived organisational support [48] and psychological safety climate [49]. Therefore our findings are in line with a prevention perspective, in which the contextual factors have the most potential for broad impacts in reducing bullying and its effects as they can be implemented in the workplace [50, 51]. Acting directly on the bullying prevention can help to reduce negative health outcomes, such as the MSDs presented here.

## Figures and Tables

**Table 1:** Means, standard deviations, percentages, Cronbach's *α*, and correlations (*N* = 512).

Variables	*N* item	M (SD)	%	*α*	1	2	3	4	5	6	7	8	9	10
(1) Age^+^	—	43.64 (7.8)	—	—	—									
(2) Gender (1 = male)^+^	—	—	37.1	—	−0.022	—								
(3) Role (1 = employee)^+^	—	—	81.6	—	−0.109^*^	−0.315^***^	—							
(4) Contract (1 = part-time)^+^	—	—	52.3	—	−0.256^***^	−0.465^***^	0.406^***^	—						
(5) Physical demand^+^	5	2.71 (0.73)	—	0.77	0.019	−0.095^*^	0.069	0.064	—					
(6) Bullying	9	1.67 (0.69)	—	0.87	0.066	0.108^*^	−0.087	−0.111^*^	0.238^***^	—				
(7) Job-related strain	5	17.30 (7.29)	—	0.78	0.157^***^	−0.105^*^	0.048	0.011	0.447^***^	0.327^***^	—			
(8) Low back (1 = yes)	—	—	74.6	—	0.015	−0.165^***^	0.129^**^	0.081	0.347^***^	0.169^***^	0.285^***^	—		
(9) Upper back (1 = yes)	—	—	53.1	—	0.090^*^	−0.162^**^	0.111^*^	0.099^*^	0.308^***^	0.214^***^	0.294^***^	0.387^***^	—	
(10) Neck (1 = yes)	—	—	60.5	—	0.084	−0.273^***^	0.113^*^	0.110^*^	0.266^***^	0.148^**^	0.263^***^	0.374^***^	0.403^***^	—
(11) Shoulders (1 = yes)	—	—	56.6	—	0.192^***^	−0.160^***^	0.053	0.049	0.351^***^	0.141^**^	0.253^***^	0.259^***^	0.363^***^	0.447^***^

Notes: ^+^confounding variables; ^***^ *P* < 0.001; ^**^ *P* < 0.01; ^*^ *P* < 0.05.

**Table 2:** Direct effects (*N* = 512).

	Mediator	Outcome variables MSD			
	Strain	Low back	Upper back	Neck	Shoulders
Bullying	2.539^***^	0.474^*^	0.534^**^	0.371^*^	0.209
Strain	—	0.046^**^	0.042^**^	0.040^*^	0.021
Physical demand	3.795^***^	0.888^***^	0.634^***^	0.524^***^	0.959^***^
Age	0.126^***^	−0.003	0.022	0.017	0.056^***^
Gender (1 = male)	−1.261	−0.671^*^	−0.506^*^	−1.169^***^	−0.571^*^
Role (1 = employee)	0.604	0.535	0.366	0.224	0.066
Contract (1 = part-time)	−0.062	−0.132	0.222	−0.042	0.108

Notes: coefficients are not standardized. ^***^ *P* < 0.001; ^**^ *P* < 0.01; ^*^ *P* < 0.05.

**Table 3:** Indirect mediation effects of work-related strain between bullying and MSDs (*N* = 512).

	Low back		Upper back		Neck		Shoulders	
	Effect	C.I.	Effect	C.I.	Effect	C.I.	Effect	C.I.
Mediation	0.117	0.025; 0.236	0.106	0.027; 0.209	0.102	0.024; 0.206	0.053	−0.030; 0.143

Notes: bootstrap confidence intervals were constructed using 5000 samples. When 95% confidence interval did not include zero, the indirect effect is significantly different between the level of zero and 0.05.
